# Evaluation of a New Chemiluminescent Immunoassay-Based Interferon-Gamma Release Assay for Detection of Latent Tuberculosis Infection

**DOI:** 10.3390/medicina59101734

**Published:** 2023-09-27

**Authors:** Keun Ju Kim, Seong-Eun Ryu, Ha-Na Lee, Seung-Hwan Oh, Chulhun L. Chang

**Affiliations:** 1 Department of Laboratory Medicine, Korea University College of Medicine, Seoul 02841, Republic of Korea; themicrobialworld@gmail.com; 2 Department of Laboratory Medicine, Pusan National University Yangsan Hospital, Yangsan 50612, Republic of Korea; paracelsus@daum.net; 3 Department of Laboratory Medicine, Pusan National University Hospital, Busan 49241, Republic of Korea; ariseyouth@naver.com; 4 Department of Laboratory Medicine, School of Medicine, Pusan National University, Yangsan 50612, Republic of Korea; leehana0114@nate.com

**Keywords:** latent tuberculosis infection, interferon-gamma release assay, diagnosis, chemiluminescent immunoassay, T-SPOT, comparison

## Abstract

*Background and Objectives*: This study aimed to evaluate the performance of a new chemiluminescent immunoassay-based tuberculosis (TB) interferon-gamma release assay (IGRA), AdvanSureI3 TB-IGRA (LG Chem Ltd., Seoul, Republic of Korea), for detecting latent tuberculosis infection in comparison with T-SPOT.*TB* (Oxford Immunotec, Oxford, UK). *Materials and Methods*: Between June 2021 and December 2021, 125 non-duplicate blood specimens were collected from adult volunteers; each subject received both tests concurrently. Total agreement and Cohen’s kappa coefficient (κ) were used to calculate concordance. The Jonckheere–Terpstra test was used to examine the correlation between interferon-gamma (IFN-γ) levels in AdvanSureI3 TB-IGRA and spot counts in T-SPOT.*TB*. *Results*: The IGRA findings of the two assays revealed 90.8% (95% confidence interval [CI] = 84.2–94.8) total agreement with κ of 0.740 (95% CI = 0.595–0.885), showing substantial agreement between the two tests. Additionally, the amount of IFN-γ in AdvanSureI3 TB-IGRA increased with the spot counts in T-SPOT.*TB* (*p* < 0.001). *Conclusions*: Our research revealed that the results of the AdvanSureI3 TB-IGRA were comparable to those of T-SPOT.*TB*.

## 1. Introduction

Tuberculosis (TB) remains one of the leading causes of death worldwide. The World Health Organization (WHO) estimates that about one-fourth of the world’s population is infected with *Mycobacterium tuberculosis*, the causative agent of TB [1]. TB is a disease that spreads through the air. The bacteria are transmitted when individuals inhale tiny droplet nuclei released by contagious individuals through activities such as coughing, speaking, singing, and sneezing [1,2]. Following exposure to *M. tuberculosis*, approximately 20–25% of individuals become infected, but in most cases, the innate immune response effectively clears *M. tuberculosis* infection [2]; 5% of patients with *M. tuberculosis* infection will develop active TB disease within two years. The host immune system controls infection in the remaining 90–95%, resulting in a latent condition. A total of 5–10% of people in this category will have TB reactivation at some point in their lives [2]. Prevention and control of TB transmission can be facilitated by detecting latent TB infection (LTBI) and treating people who are most at risk of turning their LTBI into active TB [3].

LTBI is defined by the detection of an immunological response in the absence of clinical indications of active disease [2]. A paucibacillary condition characterizes LTBI, in which the small numbers of bacilli present in infected people trigger host reactions that can be used as an alternative marker for the presence of bacilli [2]. Accurate diagnosis of LTBI is difficult due to the lack of a gold standard test [4]. Currently, the WHO advises using either a tuberculin skin test (TST) or an interferon-gamma (IFN-γ) release assay (IGRA) to test for LTBI. Specificity of TST is suboptimal due to the intradermal injection of purified protein derivative, a combination of proteins, many of which are shared by *M*. *tuberculosis*, Bacille Calmette-Guérin strain, and nontuberculous mycobacteria [4]. IGRA is a blood assay that contains *M. tuberculosis*-specific antigens to enhance specificity [4]. T-SPOT.*TB* (Oxford Immunotec, Oxford, UK) using an enzyme-linked immune spot method is one of the most widely used IGRAs [3].

In recent years, the TB diagnostic pipeline has expanded considerably in terms of the number of tests, products, or methods in development [1]. Among these are new IGRAs for detecting LTBI [1]. Recently, a new chemiluminescent-based AdvanSureI3 TB-IGRA (LG CHEM Ltd., Seoul, Republic of Korea) was developed to measure TB-specific IFN-γ in whole blood. This new diagnostic platform has several merits, including ease of use, simple test procedures, and rapid turnaround time, allowing clinical laboratories to maximize testing efficiency [5]. The platform can process up to two samples per run using incubated plasma, yielding a throughput of eight samples per hour. As with other TB-IGRA diagnostic platforms [6], basic laboratory infrastructure without specific biosafety equipment is required for LTBI detection utilizing AdvaSureI3 TB-IGRA. The objective of the current study was to compare AdvanSureI3 TB-IGRA with T-SPOT.*TB* for detecting LTBI.

## 2. Materials and Methods

### 2.1. Study Participants

This study was conducted between June 2021 and December 2021 at Pusan National University Yangsan Hospital. Individuals who visited the hospital or health care workers (HCW) were recruited for the study. For comparison, 125 non-duplicate blood samples from adult volunteers over the age of 18 years were used. Whole blood samples were obtained from each participant in 5 mL lithium heparin tubes and 1 mL of three different tubes given by the vendor: negative control (NC), TB antigens (ESAT-6, CFP-10, and TB7.7), and positive control (PC). Lithium heparin tubes were used for T-SPOT.*TB* (T-SPOT), while the three specific tubes were used for the AdvanSureI3 TB-IGRA (AdvanSureI3). This study was approved by the Institutional Review Board of Pusan National University Yangsan Hospital (03-2021-008). Written informed consent was obtained from the participants.

### 2.2. AdvanSureI3

Procedures for AdvanSureI3 were performed according to the manufacturer’s instructions. In brief, the test tubes were inverted at least 20 times to mix the samples with the TB antigens attached to the tubes. The tubes were incubated for 16–24 h at 37 °C. Following incubation, the samples were centrifuged at 2000× *g* for 5 min at room temperature, and the supernatants (plasma) were immediately used for analysis. The supernatants (50 µL) from each tube were placed into the cartridge. Three cartridges per sample were run simultaneously in the analyzer for 15 min. AdvanSureI3 uses one-step sandwich immunoassay to measure TB-specific IFN-γ. For detection, two anti-human IFN-γ antibodies were used: an anti-human IFN-γ antibody-coated magnetic particle that captures IFN-γ in the plasma sample and an acridinium-conjugated anti-human IFN-γ antibody that binds to the antibody-IFN-γ complex. The relative light unit, which correlates to the amount of IFN-γ, was utilized to measure the strength of the chemiluminescent signals. Results were reported using the manufacturer’s cutoffs: a result was positive if NC was ≤8.0 IU/mL and TB antigens−NC was 0.35 IU/mL and ≥25% of NC; a result was negative if NC was ≤8.0 IU/mL, PC−NC ≥ 0.5 IU/mL and TB antigens−NC < 0.35 IU/mL or TB antigens−NC ≥ 0.35 IU/mL and <25% of NC. Other results were deemed invalid.

### 2.3. T-SPOT

T-SPOT analysis was performed according to the manufacturer’s instructions. Briefly, peripheral blood mononuclear cells (PBMCs) were isolated from the heparin tubes, washed, and then counted. Isolated PBMCs were placed in four microplate wells subject to two different panels of *M. tuberculosis*-specific antigens (ESAT-6 and CFP-10), a phytohemagglutinin positive control, and nil control. The PBMCs were incubated for 16–24 h at 37 °C to stimulate IFN-γ secretion. Secreted cytokines were captured on the surface of the membrane using antibodies. The T-SPOT results were interpreted by subtracting the spot count in the nil control from the spot count in each of the panels. According to the manufacturer’s instructions, a positive result was obtained if panel A minus the nil control and/or panel B minus the nil control was >7 spots. A borderline result was obtained if the highest of the panel A minus the nil control or panel B minus the nil control spot count was 5, 6, or 7 spots. A negative result was obtained if both panel A minus the nil control and panel B minus the nil control was ≤4 spots. A specimen was deemed invalid if it produced more than 10 spots in the nil control or fewer than 20 spots in the positive control (unless either panel A or panel B was positive).

### 2.4. Statistical Methods

The McNemar test was used to compare the difference in LTBI detection rate between the two assays. The proportion of concordance results and Cohen’s kappa coefficient (κ) were used to measure test agreement for qualitative outcomes (0.81 ≤ κ ≤ 1.00, almost perfect; 0.61 ≤ κ ≤ 0.80, substantial; 0.41 ≤ κ ≤ 0.60, moderate; 0.21 ≤ κ ≤ 0.40, fair; 0.00 ≤ κ ≤ 0.20, slight; κ < 0.00, poor). The sensitivity and specificity of AdvanSureI3 was calculated based on the assumption that T-SPOT served as the gold-standard method, with the exception of discordant results. To address discordant results, electronic medical records of the participants were examined to assess their previous history of *M. tuberculosis* infection. Participants with a history of previous TB or LTBI were categorized as true positives. The receiver operating characteristic (ROC) curve was plotted to assess the overall diagnostic performance of AdvanSureI3. The evaluation of the area under the ROC curve (AUC) indicated different levels of performance: AUC values falling between 0.9 and 1 were classified as excellent, those between 0.8 and 0.9 as good, within 0.7–0.8 as fair, within 0.6–0.7 as poor, and between 0.5 and 0.6 as failed [7]. The Jonckheere–Terpstra test was performed to determine whether the IFN-γ levels in AdvanSureI3 increased with the spot counts in T-SPOT, which were divided into three subgroups: 0–4, 5–7, and >7. Furthermore, Spearman’s correlation test was employed to assess the correlation between these two variables. To assess factors associated with discordant results, we evaluated continuous variables for normality using the Shapiro–Wilk test. Given that the data did not follow a normal distribution, we employed the nonparametric Mann–Whitney U test for comparisons. Categorical data were compared using Fisher’s exact test. Significant variables (*p* < 0.05) were analyzed using logistic regression analysis. All *p* values were reported to three decimal places. Statistical analyses were performed using SPSS for Windows v26.0 (IBM, Armonk, NY, USA) and GraphPad Prism version 10.0.2 for Windows (GraphPad Software, Boston, MA, USA).

## 3. Results

### 3.1. Clinical Characteristics

The characteristics of the study participants are presented in Table 1. A total of 125 participants were subjected to analysis. The mean age was 49.2 years; 77.6% were female and 83.2% were HCW. No one had human immunodeficiency virus (HIV)/acquired immunodeficiency syndrome. The study population included 16 people who had LTBI and four participants had previously active TB (Table 1).

### 3.2. Diagnostic Results between AdvanSureI3 and T-SPOT

**Figure 1:**
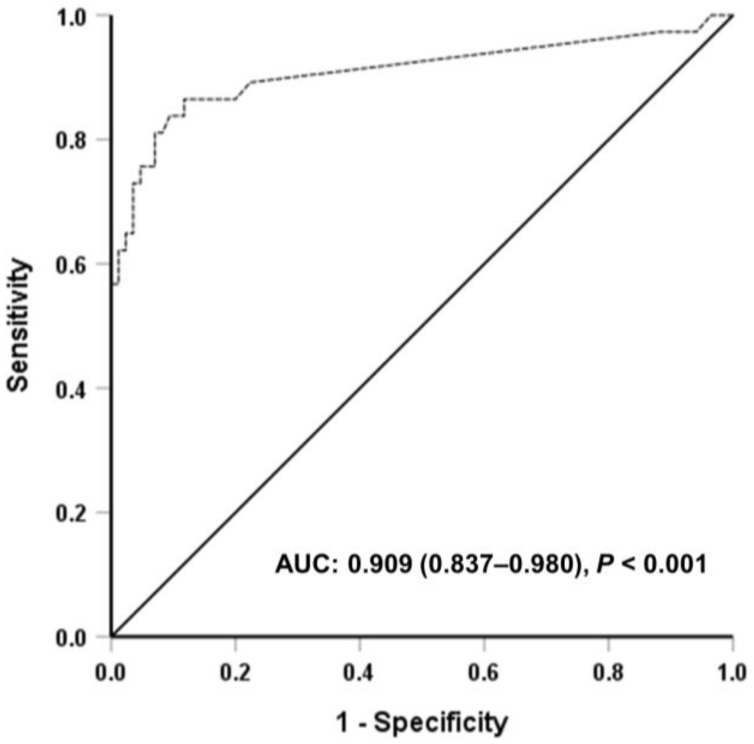
Receiver operating characteristic curve of AdvanSureI3 to diagnose latent tuberculosis infection. Abbreviation: AUC, area under the receiver operating characteristic curve (95% confidence interval).

Among all participants, 29.6% (37/125) showed positive results with one or both methods (Table 2). Of these 37 positive samples, 31 and 28 were detected by AdvanSureI3 and T-SPOT assays, respectively (*p* = 0.606) (Table 2). In total, 17 discordant IGRA results were detected; six were AdvanSureI3-negative/T-SPOT-positive, five were AdvanSureI3-positive/T-SPOT-negative, three T-SPOT-borderline, and three T-SPOT-invalid findings. The basal characteristics of 17 people with discordant IGRA results were compared to those of participants with concordant IGRA results. Age was significantly associated with discordant IGRA results (*p* = 0.004) (Table 1). Simple logistic regression analysis also showed increasing odds of the discordant results associated with age (odds ratio 1.073, 95% confidence interval 1.021–1.128, *p* = 0.005). In the 119 participants (excluding six participants with borderline and invalid findings by T-SPOT), the observed agreement between the tests was 90.8%, with substantial concordance (κ = 0.740) (Table 2). LTBI was detected using AdvanSureI3 with 81.8% sensitivity and 96.6% specificity (Table 2). The AUC value was 0.909, indicating excellent performance (Figure 1). As our cohort encompassed a total of 20 participants with a history of prior *M. tuberculosis* infection (Table S1), we also conducted an evaluation of diagnostic accuracy within this subgroup (Table S2). The results indicated similar sensitivity and specificity compared to the entire cohort. While the levels of IFN-γ in AdvanSureI3 were elevated in the group with previous *M. tuberculosis* infection as opposed to the group without such an infection (Table S3), this distinction was not evident among individuals who tested positive for AdvanSureI3 (Table S4).

### 3.3. Correlation of IFN-γ Values of AdvanSureI3 and Spot Counts of T-SPOT

**Figure 2:**
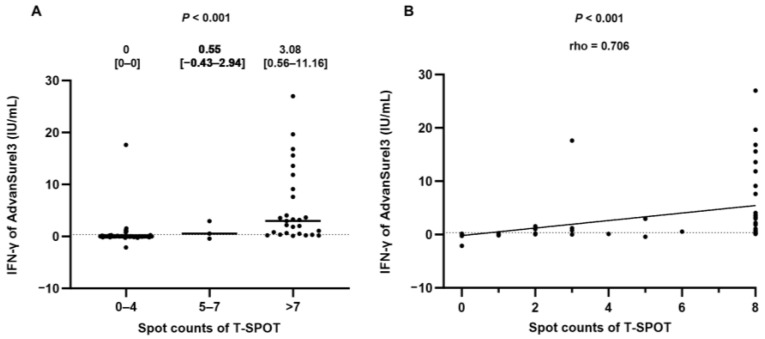
Correlation of the IFN-γ response in AdvanSureI3 and spot counts in T-SPOT. (**A**) The Jonckheere–Terpstra test was assessed between the IFN-γ response and spot counts, divided into three groups: 0–4, 5–7, and over 7 spot counts. Line indicates median, which is also represented above the plot with interquartile range. (**B**) Spearman test was performed to examine the correlation between the two variables. Dotted lines indicate the limit of positivity of AdvanSureI3 (0.35 IU/mL). An outlier (100 IU/mL of AdvanSureI3 for >7 T-SPOT) was not included for the plots.

Figure 2 depicts the correlation between the AdvanSureI3 IFN-γ levels and the spot counts of the T-SPOT that were split into three groups. As the spot counts in T-SPOT increased, there was a significant increase in IFN-γ levels in AdvanSureI3 (*p* < 0.001) (Figure 2A). The levels of IFN-γ exhibited a positive correlation with the spot counts (rho = 0.706, *p* < 0.001) (Figure 2B).

## 4. Discussion

This is the first time the new AdvanSureI3 using chemiluminescence has been compared to T-SPOT for LTBI detection. The total agreement rate and kappa value among all participants were 90.8% and 0.740, respectively, indicating a substantial degree of agreement. Despite variation in the interpretive readout, our agreement was comparable to other studies comparing the performance of QuantiFeron (Qiagen, Germantown, MD, USA) using enzyme-linked immunosorbent assay and T-SPOT for LTBI detection [8,9,10]. In addition, a significant correlation was observed in the current study between the IFN-γ levels in AdvanSureI3 and the spot counts in T-SPOT, which was comparable to the finding of the previous study [9]. The AUC analysis indicated the excellent performance of AdvanSureI3, consistent with the previous finding [5]. These results highlight that qualitative results and IFN-γ responses were comparable between the two assays.

Discordant results could be explained by a few factors [10]. Firstly, the main difference between the two methods was the specimen used (whole blood versus mononuclear cells). AdvanSureI3 quantifies the concentration of IFN-γ, while T-SPOT measures the total number of T cells that secrete IFN-γ. Hence, it is possible that a small number of activated T cells can produce a high amount of IFN-γ. Secondly, the discrepancy could be attributable to how *M. tuberculosis*-specific peptides are used in the two procedures; AdvanSureI3 mixes TB7.7, ESAT-6, and CFP-10 in a single tube, whereas T-SPOT employs ESAT-6 and CFP-10 in separate tubes. Previous studies have demonstrated that a higher proportion of discordant results between IGRAs was observed in older people vs. adults [11,12,13,14]. In our study, we also found that age was associated with the discordance between the two assays. Hence, careful interpretation of IGRA results in older people is necessary.

The WHO’s New Diagnostic Working Group recently established a framework for the evaluation of new tests for LTBI [15]. According to the framework, any new LTBI test based on concepts similar to TST and IGRA is not anticipated to significantly improve predictive performance. They advise using sensitivity and specificity or concordance compared with at least one of the currently available IGRA tests that have been endorsed by the WHO because it is difficult to conduct a study to measure predictive value. In our study, we employed T-SPOT endorsed by the WHO as a comparator and concordance as a primary endpoint for a comparative analysis of AdvanSureI3 for LTBI in compliance with the WHO guideline [15].

This study had several limitations. First, due to the small sample size drawn from a single study site, only a small number of people with prior TB and LTBI were included. Likewise, the research participants were healthy volunteers, resulting in the exclusion of subjects with HIV. Those individuals living with untreated LTBI and HIV have a higher propensity to progress to active TB compared to those without HIV infection. Consequently, it would have been advantageous to observe data pertaining to this particular group.

## 5. Conclusions

Our study showed that the results of AdvanSureI3 were comparable to those of T-SPOT for detecting LTBI. Achieving convenience without the need for technical difficulties is one of the main issues for LTBI diagnosis. Future research is warranted to examine the operational merits of AdvanSureI3.

## Figures and Tables

**Table 1:** Baseline characteristics of participants.

	All Subjects	Concordance	Discordance	
Characteristic	*N* = 125 (%)	*N* = 108 (%)	*N* = 17 (%)	*p* Value
Age (mean ± SD)	49.2 ± 13.1	48.0 ± 13.4	58.4 ± 7.8	0.004
Woman	97 (77.6)	82 (75.9)	15 (88.2)	0.357
Health care worker	104 (83.2)	90 (83.3)	14 (82.4)	1.000
Body mass index kg/m^2^, (mean ± SD)	23.6 ± 3.1	23.5 ± 3.2	24.2 ± 2.1	0.319
Hypertension	19 (15.2)	17 (15.7)	2 (11.8)	1.000
Diabetes mellitus	7 (5.6)	5 (4.6)	2 (11.8)	0.242
Solid cancer	6 (4.8)	4 (3.7)	2 (11.8)	0.188
Chronic lung disease (bronchiectasis, asthma, or allergy)	3 (2.4)	3 (2.8)	0 (0)	1.000
HIV/AIDS	0 (0)	0 (0)	0 (0)	-
Hypothyroidism	2 (1.6)	2 (1.9)	0 (0)	1.000
Chronic liver disease	4 (3.2)	3 (2.8)	1 (11.8)	0.447
Chronic kidney disease	2 (1.6)	2 (1.9)	0 (0)	1.000
Rheumatic disease	2 (1.6)	2 (1.9)	0 (0)	1.000
LTBI ^a^	16 (12.8)	12 (11.1)	4 (23.5)	0.232
Previous TB ^b^	4 (3.2)	3 (2.8)	1 (11.8)	0.447

^a^ LTBI group reveals that participants had prior positive IGRA results with no clinical symptoms. ^b^ Previous TB group shows that participants had prior active TB, underwent TB medication, and presently had no clinical and radiological evidence of active TB. Abbreviations: SD, standard deviation; HIV, human immunodeficiency virus; AIDS, acquired immunodeficiency syndrome; LTBI, latent tuberculosis infection; TB, tuberculosis; IGRA, interferon-gamma release assay.

**Table 2:** Qualitative results between AdvanSureI3 and T-SPOT.

AdvanSureI3	T-SPOT						
	Positive	Negative	Borderline	Invalid	Total	Overall Agreement	Kappa
Positive	22 (17.6%)	5 ^a^ (4.0%)	2 ^b^ (1.6%)	2 ^c^ (1.6%)	31 (24.8%)	90.8% (84.2–94.8) ^e^	0.740 (0.595–0.885)
Negative	6 ^d^ (4.8%)	86 (68.8%)	1 (0.8%)	1 (0.8%)	94 (75.2%)		
Invalid	0 (0%)	0 (0%)	0 (0%)	0 (0%)	0 (0%)		
Total	28 (22.4%)	91 (72.8%)	3 (2.4%)	3 (2.4%)	125 (100%)		
Sensitivity = 81.8% (27/33) with 95% CI: 65.6–91.4%							
Specificity = 96.6% (86/89) with 95% CI: 90.5–99.1%							

^a^ Two of these discordant results were considered true positive because of prior LTBI or TB history. ^b^ Two borderline results were considered true positive because of prior LTBI or TB history. ^c^ One of these invalid results was considered true positive because of prior LTBI or TB history. ^d^ These T-SPOT-only positive results were considered true positive for sensitivity and specificity calculations. ^e^ 95% confidence interval (CI).

## Data Availability

In the article, all data pertinent to the study are included.

## Supplementary Materials

The following supporting information can be downloaded at: https://www.mdpi.com/article/10.3390/medicina59101734/s1, Table S1: Raw data of IFN-γ levels of AdvanSureI3 and spot counts of T-SPOT for individuals with previous TB or LTBI; Table S2: Qualitative results between AdvanSureI3 and T-SPOT for individuals with previous TB or LTBI; Table S3: Comparison of IFN-γ levels between individuals with or without previous TB or LTBI; Table S4: Comparison of IFN-γ levels between AdvanSureI3-positive individuals with or without previous TB or LTBI.

## References

[1] World Health Organization (2022). Global Tuberculosis Report 2022: WHO. https://www.who.int/teams/global-tuberculosis-programme/tb-reports/global-tuberculosis-report-2022.

[2] Goletti D., Delogu G., Matteelli A., Migliori G.B. (2022). The role of IGRA in the diagnosis of tuberculosis infection, differentiating from active tuberculosis, and decision making for initiating treatment or preventive therapy of tuberculosis infection. Int. J. Infect. Dis..

[3] Petruccioli E., Farroni C., Cuzzi G., Vanini V., Palmieri F., Vittozzi P., Goletti D. (2022). VIDAS^®^ TB-IGRA reagents induce a CD4^+^ and CD8^+^ T-cell IFN-γ response for both TB infection and active TB. Int. J. Tuberc. Lung Dis..

[4] Venkatappa T.K., Punnoose R., Katz D.J., Higgins M.P., Banaei N., Graviss E.A., Belknap R.W., Ho C.S. (2019). Comparing QuantiFERON-TB Gold Plus with Other Tests to Diagnose Mycobacterium tuberculosis Infection. J. Clin. Microbiol..

[5] Kim J.J., Park Y., Choi D., Kim H.S. (2020). Performance Evaluation of a New Automated Chemiluminescent Immunoanalyzer-Based Interferon-Gamma Releasing Assay AdvanSure I3 in Comparison with the QuantiFERON-TB Gold In-Tube Assay. Ann. Lab. Med..

[6] World Health Organization (2008). Stop TB Partnership Retooling Task Force and New Diagnostic Working Group. New Laboratory Diagnostic Tools for Tuberculosis Control.

[7] Nahm F.S. (2022). Receiver operating characteristic curve: Overview and practical use for clinicians. Korean J. Anesthesiol..

[8] Kim T.Y., Chang H.E., Lee S.W., Seo S.H., Hong Y.J., Park J.S., Park K.U. (2019). A novel strategy for interpreting the T-SPOT.TB test results read by an ELISPOT plate imager. PLoS ONE.

[9] Takeda K., Nagai H., Suzukawa M., Sekiguchi R., Akashi S., Sato R., Narumoto O., Kawashima M., Suzuki J., Ohshima N. (2020). Comparison of QuantiFERON-TB Gold Plus, QuantiFERON-TB Gold In-Tube, and T-SPOT.TB among patients with tuberculosis. J. Infect. Chemother..

[10] Du F., Xie L., Zhang Y., Gao F., Zhang H., Chen W., Sun B., Sha W., Fang Y., Jia H. (2018). Prospective Comparison of QFT-GIT and T-SPOT.TB Assays for Diagnosis of Active Tuberculosis. Sci. Rep..

[11] Kobashi Y., Sugiu T., Shimizu H., Ohue Y., Mouri K., Obase Y., Miyashita N., Oka M. (2009). Clinical evaluation of the T-SPOT.TB test for patients with indeterminate results on the QuantiFERON TB-2G test. Intern. Med..

[12] Ferrara G., Losi M., D’Amico R., Cagarelli R., Pezzi A.M., Meacci M., Meccugni B., Marchetti Dori I., Rumpianesi F., Roversi P. (2009). Interferon-gamma-release assays detect recent tuberculosis re-infection in elderly contacts. Int. J. Immunopathol. Pharmacol..

[13] Bae W., Park K.U., Song E.Y., Kim S.J., Lee Y.J., Park J.S., Cho Y.J., Yoon H.I., Yim J.J., Lee C.T. (2016). Comparison of the Sensitivity of QuantiFERON-TB Gold In-Tube and T-SPOT.TB According to Patient Age. PLoS ONE.

[14] Scordo J.M., Aguillón-Durán G.P., Ayala D., Quirino-Cerrillo A.P., Rodríguez-Reyna E., Joya-Ayala M., Mora-Guzmán F., Ledezma-Campos E., Villafañez A., Schlesinger L.S. (2021). Interferon gamma release assays for detection of latent Mycobacterium tuberculosis in older Hispanic people. Int. J. Infect. Dis..

[15] World Health Organization (2020). Framework for the Evaluation of New Tests for Tuberculosis Infection.

