# Supplementary material for: Evaluation of a New Chemiluminescent Immunoassay-Based Interferon-Gamma Release Assay for Detection of Latent Tuberculosis Infection
Source: Medicina (Kaunas). 2023 Sep 27;59(10):1734. doi: 10.3390/medicina59101734 (PMC10608617; doi:10.3390/medicina59101734)
Supplement: Supplementary file 1 [file medicina-59-01734-s001.zip › medicina-2610981-supplementary.docx]

**Table S1.** Raw data of IFN-γ levels of AdvanSureI3 and spot counts of T-SPOT for individuals with previous TB or LTBI

|  |  |  | | AdvanSureI3 results (IFN-γ levels) | | | | |  | T-SPOT results (# of spot) | | | | |
| --- | --- | --- | --- | --- | --- | --- | --- | --- | --- | --- | --- | --- | --- | --- |
| Participants | *M. tuberculosis* infection | |  | Interpretation | NC  (IU/mL) | TB  (IU/mL) | PC  (IU/mL) | TB−NC  (IU/mL) |  | Nil | ESAT-6  panel A | CFP-10  panel B | PC | Interpretation |
| 1 | previous TB | |  | Positive | < 0.00 | 15.59 | 30.76 | 15.59 |  | 0 | ≥ 8 | ≥ 8 | ≥ 20 | Positive |
| 2 | LTBI | |  | Positive | 0.45 | 2.62 | 5.70 | 2.17 |  | 0 | ≥ 8 | 0 | ≥ 20 | Positive |
| 3 | LTBI | |  | Positive | < 0.00 | 9.11 | 10.05 | 9.11 |  | 0 | ≥ 8 | ≥ 8 | ≥ 20 | Positive |
| 4 | LTBI | |  | Positive | < 0.00 | 2.94 | 12.22 | 2.94 |  | 0 | 1 | 5 | ≥ 20 | Borderline |
| 5 | LTBI | |  | Positive | < 0.00 | 0.83 | 22.74 | 0.83 |  | 0 | 5 | ≥ 8 | ≥ 20 | Positive |
| 6 | LTBI | |  | Positive | < 0.00 | 13.57 | 41.71 | 13.57 |  | 0 | ≥ 8 | ≥ 8 | ≥ 20 | Positive |
| 7 | LTBI | |  | Positive | < 0.00 | 2.00 | 13.78 | 2.00 |  | 0 | ≥ 8 | ≥ 8 | ≥ 20 | Positive |
| 8 | LTBI | |  | Positive | < 0.00 | 3.17 | 7.51 | 3.17 |  | 0 | ≥ 8 | 3 | ≥ 20 | Positive |
| 9 | previous TB | |  | Negative | < 0.00 | 0.13 | 67.52 | 0.13 |  | 0 | ≥ 8 | 2 | ≥ 20 | Positive |
| 10 | previous TB | |  | Positive | < 0.00 | 2.99 | > 100.00 | 2.99 |  | 0 | ≥ 8 | ≥ 8 | ≥ 20 | Positive |
| 11 | LTBI | |  | Negative | 0.16 | < 0.00 | 37.65 | −0.16 |  | 0 | 1 | 1 | ≥ 20 | Negative |
| 12 | LTBI | |  | Positive | < 0.00 | 0.64 | 32.12 | 0.64 |  | 0 | ≥ 8 | 0 | ≥ 20 | Positive |
| 13 | LTBI | |  | Positive | 0.07 | 19.72 | 58.94 | 19.65 |  | 0 | 8 | ≥ 8 | ≥ 20 | Positive |
| 14 | LTBI | |  | Positive | < 0.00 | 29.71 | 18.37 | 29.71 |  | ≥ 10 | ≥ 8 | ≥ 8 | ≥ 20 | Invalid |
| 15 | LTBI | |  | Negative | < 0.00 | 0.20 | 13.94 | 0.20 |  | 0 | ≥ 8 | ≥ 8 | ≥ 20 | Positive |
| 16 | LTBI | |  | Positive | < 0.00 | 1.19 | 53.00 | 1.19 |  | 0 | 1 | 2 | ≥ 20 | Negative |
| 17 | LTBI | |  | Positive | 0.38 | 17.98 | 76.63 | 17.60 |  | 0 | 3 | 0 | ≥ 20 | Negative |
| 18 | previous TB | |  | Positive | < 0.00 | 1.86 | 11.65 | 1.86 |  | 0 | ≥ 8 | 0 | ≥ 20 | Positive |
| 19 | LTBI | |  | Positive | < 0.00 | 16.82 | >100.00 | 16.82 |  | 0 | ≥ 8 | 1 | ≥ 20 | Positive |
| 20 | LTBI | |  | Positive | < 0.00 | 11.84 | >100.00 | 11.84 |  | 0 | ≥ 8 | 0 | ≥ 20 | Positive |

Abbreviations: IFN-γ, interferon-gamma; NC, negative control; PC, positive control; TB, tuberculosis; LTBI, latent tuberculosis infection.

**Table S2.** Qualitative results between AdvanSureI3 and T-SPOT for individuals with previous TB or LTBI

| AdvanSureI3 | T-SPOT | | | |  |  |  |
| --- | --- | --- | --- | --- | --- | --- | --- |
|  | Positive | Negative | Borderline | Invalid | Total | Overall  agreement | Kappa |
| Positive | 13 | 2^a^ | 1^a^ | 1^a^ | 17 | 77.8%  (54.8–91.0)^b^ | 0.200  (−0.351–0.751) |
| Negative | 2^a^ | 1 | 0 | 0 | 3 |  |  |
| Invalid | 0 | 0 | 0 | 0 | 0 |  |  |
| Total | 15 | 3 | 1 | 1 | 20 |  |  |
| Sensitivity = 89.5% (17/19) with 95% CI: 68.6%–98.1% | | | | | | | |
| Specificity = 100.0% (1/1) with 95% CI: 5.1%–100.0% | | | | | | | |

^a^These discordant results were considered true positive because of previous TB or LTBI history. ^b^95% confidence interval (CI).

Abbreviations: TB, tuberculosis; LTBI, latent tuberculosis infection.

**Table S3.** Comparison of IFN-γ levels between individuals with or without previous TB or LTBI

| IFN-γ of AdvanSureI3 | Total individuals | Individuals without TB or LTBI | Individuals with TB or LTBI | *p* Value^a^ |
| --- | --- | --- | --- | --- |
| Positivity, % (n) | 24.8% (31/125) | 13.3% (14/105) | 85.0% (17/20) | < 0.001 |
| IFN-γ levels, median [IQR] | 0.00 [0.00–0.44] | 0.00 [0.00–0.07] | 2.97 [0.92–15.09] | < 0.001 |

^a^Categorical variable was compared using Fisher’s exact test, while the continuous variable was compared using the Mann–Whitney U test after the Shapiro–Wilk test.

Abbreviations: IFN-γ, interferon-gamma; TB, tuberculosis; LTBI, latent tuberculosis infection; IQR, interquartile range.

**Table S4.** Comparison of IFN-γ levels between AdvanSureI3-positive individuals with or without previous TB or LTBI

| IFN-γ of AdvanSureI3 | Total individuals | Individuals without TB or LTBI | Individuals with TB or LTBI | *p* Value^a^ |
| --- | --- | --- | --- | --- |
| Positivity, % (n) | 100.0% (31/31) | 100.0% (14/14) | 100.0% (17/17) | - |
| IFN-γ levels, median [IQR] | 3.17 [1.19–13.57] | 2.41 [0.97–4.95] | 3.17 [1.93–16.20] | 0.234 |

^a^The continuous variable was compared using the Mann–Whitney U test after the Shapiro–Wilk test.

Abbreviations: IFN-γ, interferon-gamma; TB, tuberculosis; LTBI, latent tuberculosis infection; IQR, interquartile range
